# Supplementary material for: Urban and rural prevalence of tuberculosis in low- and middle-income countries: A systematic review and meta-analysis
Source: PLoS Med. 2026 Apr 6;23(4):e1004779. doi: 10.1371/journal.pmed.1004779 (PMC13068319; doi:10.1371/journal.pmed.1004779)
Supplement: S1 Table — Summary of inclusion and exclusion eligibility criteria. (DOCX) [file pmed.1004779.s002.docx]

**S1 Table:** Inclusion and exclusion eligibility criteria

|  | **Include** | **Exclude** |
| --- | --- | --- |
| **Population** | Adults (≥ 15 years) or all-age samples from low- and middle-income countries (LMICs) as defined by the World Bank classification (2022-2023)    Studies allowing stratified analysis of active TB prevalence by sex (refer to this for full-text screening only) | Only includes symptomatic or healthcare- seeking individuals. Healthcare seeking can be for any disease.    Studies focused solely on occupational or university contexts.    Studies looking at latent TB prevalence.    Studies on congregate setting (occupational, prison, health facility, homeless shelter, etc.)  Research conducted in high-income countries.    Only children (< 15 years) |
| **Comparator/ Context** | TB disease prevalence    Total TB Prevalence: This includes all TB cases that are substantiated through bacteriological, radiological, or clinical evidence, capturing the full spectrum of the disease presentation.    Bacteriologically Confirmed TB: This subset is restricted to TB cases that have been confirmed through bacteriological methods such as smear microscopy, culture, or molecular diagnostics (e.g., Xpert MTB/RIF). Where the data allow, these cases will be further stratified into smear-positive and smear-negative.    TB Without Bacteriological Confirmation: This classification is reserved for TB cases diagnosed on the basis of radiological or clinical evidence in the absence of bacteriological confirmation. | Only extra-pulmonary TB (EPTB) due to the specialist nature of diagnosis.    Only DR-TB    Only latent TB infection prevalence |
| **Outcome** | Studies allowing stratified analysis of active TB prevalence by at least one of sex, urban/rural location, HIV status, and age group (refer to this for full-text screening only) | Unstratified active TB prevalence estimates. |
| **Study characteristics** | Prevalence surveys, whether stand-alone or part of larger studies (case-control, cohort studies, RCTs)  Studies with national to sub-national coverage | Routine notification or facility-based reporting  Contact tracing studies  Modelling studies |
