# Supplementary figures and images for: Urban and rural prevalence of tuberculosis in low- and middle-income countries: A systematic review and meta-analysis
Source: PLoS Med. 2026 Apr 6;23(4):e1004779. doi: 10.1371/journal.pmed.1004779 (PMC13068319; doi:10.1371/journal.pmed.1004779)

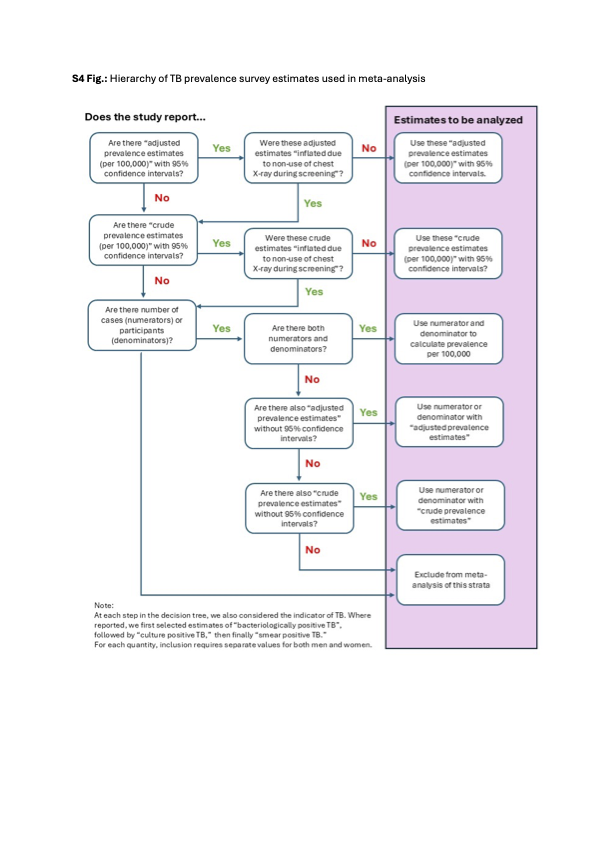

Supplement: S1 Fig — Summary of hierarchical approach to use of study prevalence estimates for inclusion in meta-analysis. (TIFF) [file pmed.1004779.s004.tiff]

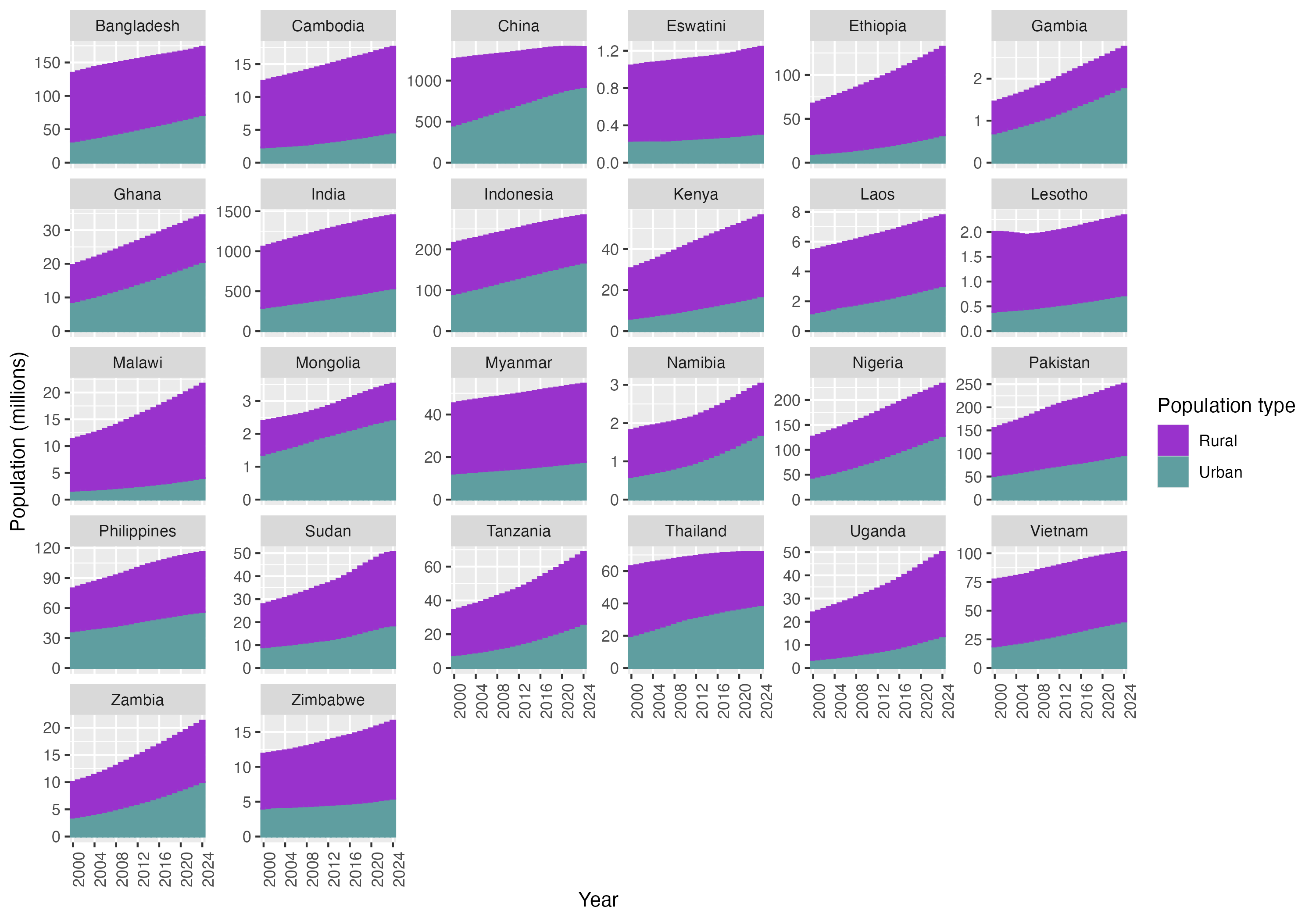

Supplement: S2 Fig — Urban and rural population data obtained from United Nations Populations 2024 (https://population.un.org/wpp/). (TIFF) [file pmed.1004779.s008.tiff]

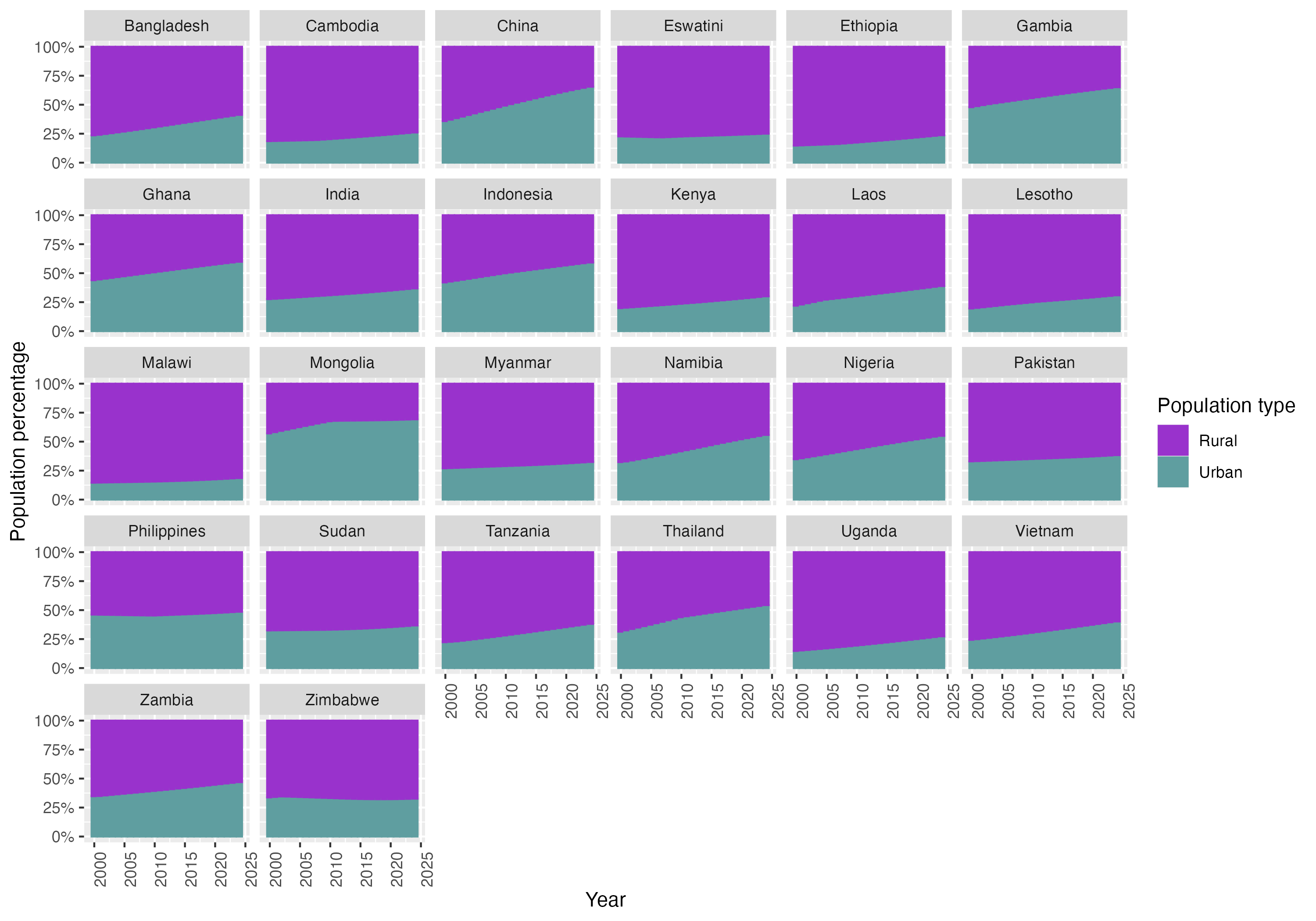

Supplement: S3 Fig — Urban and rural population data obtained from United Nations Populations 2024 (https://population.un.org/wpp/). (TIFF) [file pmed.1004779.s009.tiff]

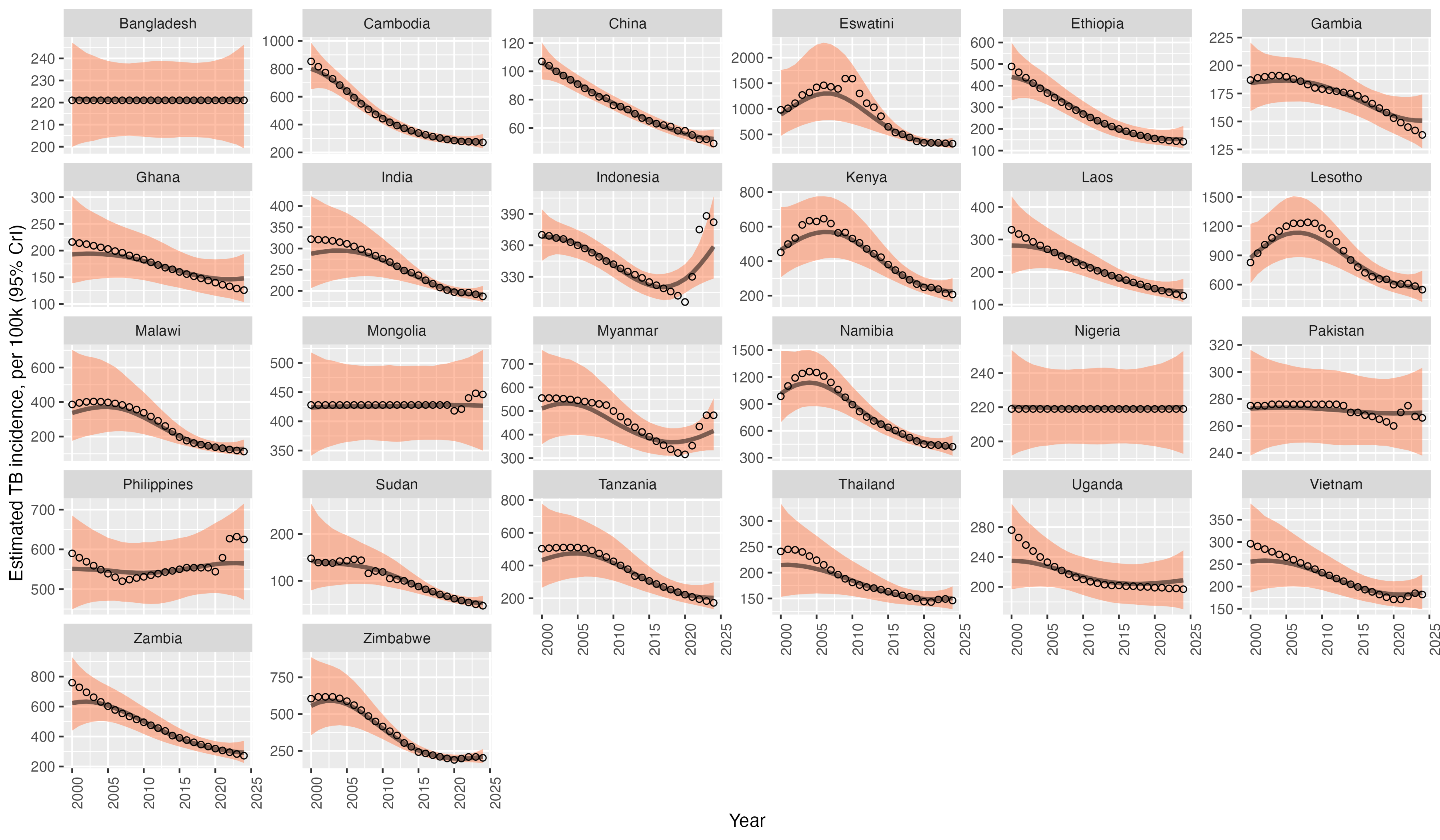

Supplement: S4 Fig — Black circles are central estimates, reported by WHO; blue line and bands are estimated from a Bayesian multivariate regression model of incidence and case detection ratio data. (TIFF) [file pmed.1004779.s010.tiff]

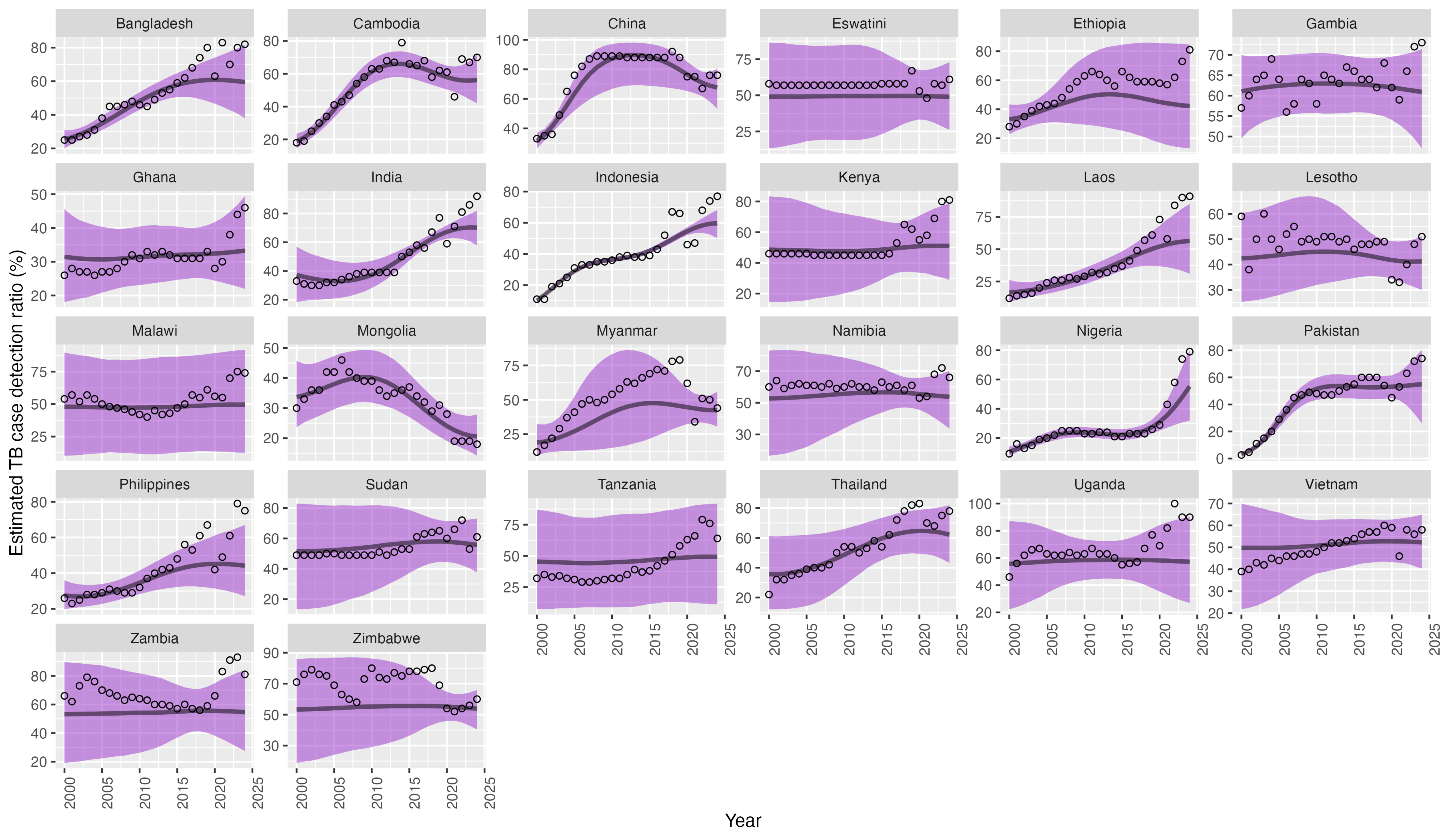

Supplement: S5 Fig — Black circles are central estimates, reported by WHO; purple line and bands are estimated from a Bayesian multivariate regression model of incidence and case detection ratio data. (TIFF) [file pmed.1004779.s011.tiff]

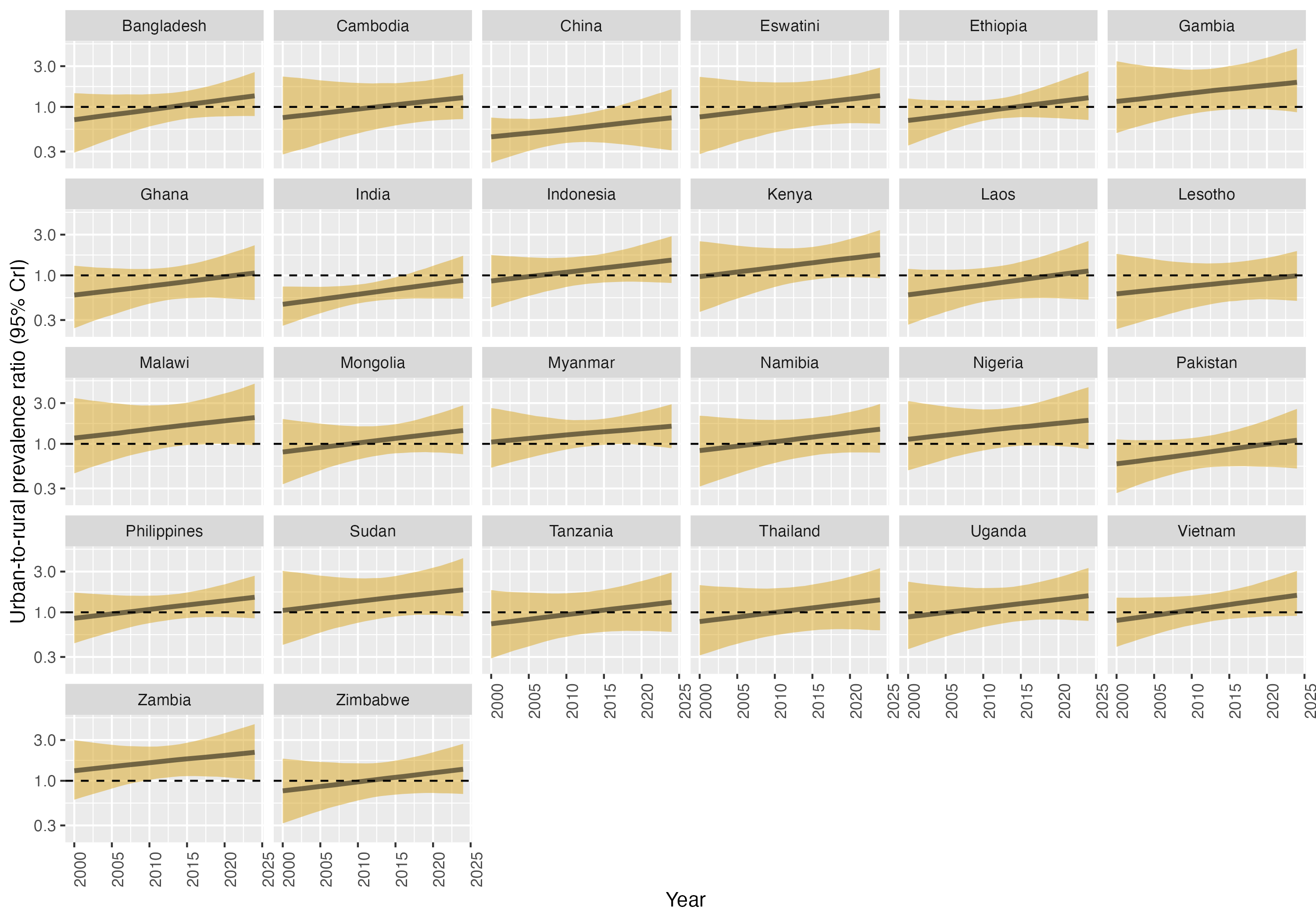

Supplement: S6 Fig — Country-specific predictions of time trends in urban-to-rural bacteriologically-confirmed TB, estimated from a Bayesian meta-analysis model (TIFF) [file pmed.1004779.s012.tiff]

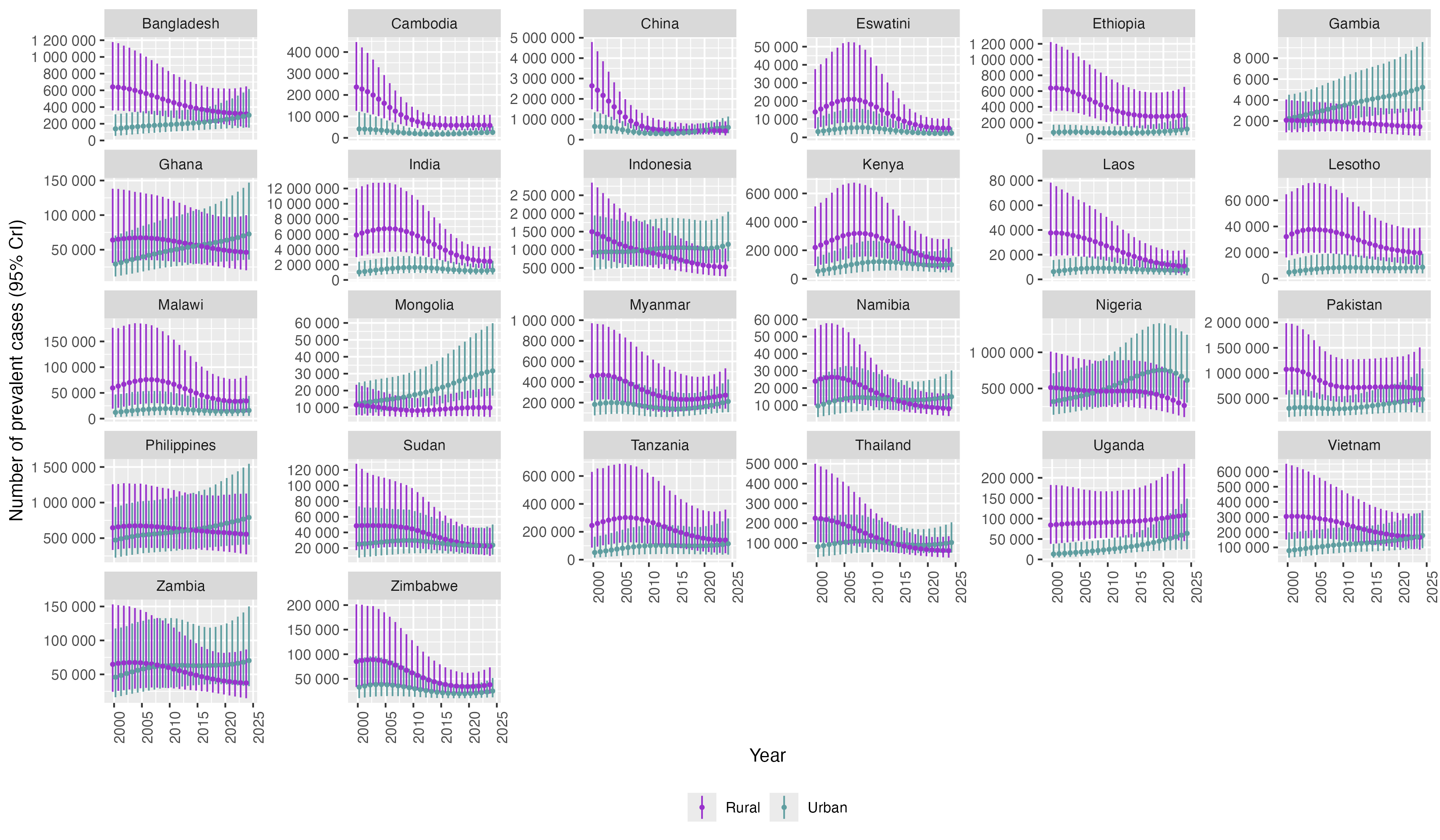

Supplement: S7 Fig — Estimated from a Bayesian multivariate regression model, which jointly estimated TB incidence and case detection ratio, and combined with assumptions around duration of infectiousness, distribution of urban and rural populations, with modelled estimates of urban and rural TB prevalence by year. (TIFF) [file pmed.1004779.s014.tiff]
