# Supplementary material for: Urban and rural prevalence of tuberculosis in low- and middle-income countries: A systematic review and meta-analysis
Source: PLoS Med. 2026 Apr 6;23(4):e1004779. doi: 10.1371/journal.pmed.1004779 (PMC13068319; doi:10.1371/journal.pmed.1004779)
Supplement: S1 Text — Description of additional statistical methods. (DOCX) [file pmed.1004779.s005.docx]

**S1 Text:** Supplemental methods for estimating urban-to-rural prevalence ratio, and prevalence and burden of TB in urban and rural populations

**Statistical modelling of urban-to-rural population ratio for burden estimation**

To estimate the urban-to-rural TB prevalence burden (bacteriologically-confirmed), we fitted a Bayesian meta-analysis model to data extracted from 46 TB prevalence surveys from 26 countries conducted between 2000 and 2024. In models estimating global, and pooled regional prevalence ratios, we removed two countries from the Eastern Mediterranean Region with disparate estimates. For each survey $s$, conducted in country $c$ (or WHO region $r$ in regional analyses), we calculated the log odds ratio of TB prevalence comparing urban to rural populations, $y_{s}$, with associated standard error $\sigma_{s}$, approximated from adjusted or unadjusted 95% confidence intervals. Where confidence intervals were unavailable, standard errors were approximated using a normal approximation to the binomial variance.

We assumed log odds ratios were normally distributed around their expected values:

$$y_{s} \sim\mathcal{N(}\mu_{s},\sigma_{s}^{2})$$

### Main (global) analysis: surveys nested within countries

For the main analysis, surveys were nested within countries, allowing for both between-country heterogeneity and within-country correlation across surveys:

$$\mu_{s}=\alpha+\beta\text{ }\text{year}_{s}+u_{c}+v_{c}\text{ }\text{year}_{s}+w_{s(c)}$$

where:

- $\alpha$ is the overall intercept
- $\beta$ is the fixed effect of calendar year
- $u_{c}\mathcal{\sim N}(0,\sigma_{u}^{2})$ is a country-specific random intercept
- $v_{c}\mathcal{\sim N}(0,\sigma_{v}^{2})$ is a country-specific random slope for year
- $w_{s(c)}\mathcal{\sim N}(0,\sigma_{w}^{2})$ is a survey-level random intercept nested within country

This formulation explicitly accounts for correlation between multiple surveys conducted within the same country.

### Regional pooled analyses: surveys nested within WHO regions

For regional pooled analyses, surveys were instead nested within WHO regions, replacing the country-level random effects with region-level terms:

$$\mu_{s}=\alpha_{r}+\beta_{r}\text{ }\text{year}_{s}+u_{r}+v_{r}\text{ }\text{year}_{s}+w_{s(r)}$$

where $u_{r}$ and $v_{r}$ are region-specific random intercepts and slopes, and $w_{s(r)}$ is a survey-level random intercept nested within WHO region.

And weakly informative priors:

- $\beta0 \sim Student-t(7, 0, 1.5)$
- $\beta1 \sim Normal(0, 10)$
- $\sigma\sim Exponential(2)$

Posterior summaries are reported as posterior means with 95% credible intervals. Survey-, country-, region-, and year-specific urban-to-rural prevalence ratios were obtained by exponentiating posterior draws of $\mu_{s}$.

**Statistical modelling of incidence and case detection**

To estimate TB burden over time, we modelled national TB incidence and case detection ratios using WHO Global TB Database estimates. Projection analyses were restricted to countries with WHO incidence estimates not under review; the Democratic People’s Republic of Korea was excluded in line with the most recent WHO Global TB Report, where incidence estimates are reported to be under review.

For each country $i$ and year $t$ (2000–2024), we extracted:

- Estimated TB incidence rates per 100,000 population (median and 95% uncertainty interval)
- Estimated case detection ratios (median and 95% uncertainty interval)

National population denominators and urban–rural population fractions were obtained from the World Bank and UN Population Prospects.

These data were merged to create country-year records of TB incidence, CDR, and population structure.

### Joint incidence–case detection model

Incidence and case detection were modelled jointly using a Bayesian multivariate regression framework implemented in **brms**, with flexible non-linear temporal trends specified using Gaussian process smooths. Surveys informing urban–rural differentials were nested within countries throughout all projection analyses, ensuring consistency between prevalence ratio estimation and burden projection.

For country $i$ in year $t$:

**Incidence model**

$$\log(I_{it}\mathcal{)\sim N(}\eta_{it}^{\left( I \right)},\sigma_{it}^{\left( I \right)})$$

$$\eta_{it}^{\left( I \right)}=\alpha^{\left( I \right)}+f_{i}^{\left( I \right)}(t)+b_{i}^{\left( I \right)}$$

**Case detection model**

$$\text{logit}(\text{CDR}_{it}\mathcal{)\sim N(}\eta_{it}^{\left( C \right)},\sigma_{it}^{\left( C \right)})$$

$$\eta_{it}^{\left( C \right)}=\alpha^{\left( C \right)}+f_{i}^{\left( C \right)}(t)+b_{i}^{\left( C \right)}$$

where:

- $f_{i}^{\left( I \right)}(t)$ and $f_{i}^{\left( C \right)}(t)$ are country-specific Gaussian process smooths over calendar year
- $b_{i}^{\left( I \right)}$ and $b_{i}^{\left( C \right)}$ are country-level random intercepts
- Observation-level standard deviations $\sigma_{it}^{\left( I \right)}$ and $\sigma_{it}^{\left( C \right)}$ were derived from WHO uncertainty intervals

Weakly informative priors were used for intercepts and random effects, with default priors for Gaussian process hyperparameters.

## **Estimating prevalence and urban–rural burden**

Posterior predictions of incidence and case detection were converted into prevalence using standard disease-duration assumptions. National prevalence estimates were then apportioned to urban and rural populations using country-year-specific population fractions and posterior draws from the estimated urban-to-rural prevalence ratio distribution.

For country $i$ in year $t$:

$$P_{it}^{\left( U \right)}=P_{it}\times\pi_{it}^{\left( U \right)}\times R_{it}$$

$$P_{it}^{\left( R \right)}=P_{it}\times\pi_{it}^{\left( R \right)}$$

where $\pi_{it}^{\left( U \right)}$ and $\pi_{it}^{\left( R \right)}$ are the urban and rural population fractions, and $R_{it}$ is a posterior draw from the country-specific urban-to-rural prevalence ratio distribution.

**Model fitting**

Models were fit in brms with 4 chains, each of 4,000 iterations. Posterior predictive checks confirmed good model fit, with Gaussian process terms capturing non-linear temporal variation in both incidence and case detection across countries.
