# Supplementary material for: Urban and rural prevalence of tuberculosis in low- and middle-income countries: A systematic review and meta-analysis
Source: PLoS Med. 2026 Apr 6;23(4):e1004779. doi: 10.1371/journal.pmed.1004779 (PMC13068319; doi:10.1371/journal.pmed.1004779)
Supplement: Attachment — Submitted filename: mortazavi.pdf [file pmed.1004779.s016.pdf]

This paper reports a systematic review with meta–analysis of studies of rural–urban differences in TB prevalence in low– and middle income countries.

I was asked for a methodological report and I interpret that to include all aspects of the design and conduct of the study.

# Points of detail

**Page 5** The authors have chosen to only include studies published in English. The message I draw from Grégoire et al. (1995); Moher et al. (1996); Egger et al. (1997); Jüni et al. (2002); Konno et al. (2020); Nussbaumer-Streit et al. (2020); Bachelet et al. (2021) is that bias is possible although it is hard to predict in advance the extent or direction of it. I would not necessarily commit the authors to translate everything but at least we should know what we are missing.

**Page 5** The authors refer to limited translation resources. In a small study Jackson et al. (2019) reported good results in evidence synthesis using Google Translate. DeepL is another possibility.

**Page 6** That is a very clear report of the screening and abstraction process and the precautions taken against bias.

**Page 7** Does the statement 'potential predictors included' mean that there were others which have not been reported? This is important if others were considered but finally discarded.

**Page 8** Do the authors mean a multivariate model for incidence and case detection data, that is to say, a model with a vector–valued outcome, or do they mean a multivariable model? They use multivariable correctly elsewhere so I assume multivariate is meant here.

**Page 9** Providing proper citation for R packages as here gives authors appropriate credit for their work. (Disclaimer: I am a CRAN author although of different packages.)

**Page 10, Figure 1** Studies assessed 201, studies excluded 86, studies included 45, $201 - 86 \neq 45$. Is this explained by the slightly cryptic note in the caption about multiple reporting? If so how was the decision made as to which to include?

**Page 12** There is a dilemma here between (a) choosing to use an established classification (WHO regions) which has advantages of being defined independently of the authors and (b) using a sensible but ad hoc one. I have no particular problem with most of the WHO regions but their

idea of the Eastern Mediterranean is not mine. I do not see many common features between Sudan and Pakistan. I think most people would say Sudan is in Africa and Pakistan is in South Asia. The inclusive approach would be to allocate them to Africa and South East Asia respectively and explain why the WHO regions have had to be modified.

**Page 12** Comparison between the regions would be clearer if we also saw estimates of the relative prevalence ratio with a credible interval.

**Page 14** Although it is perfectly true that 2.2% credible interval -2.5 to 8.0 is consistent with a small increase it is also consistent with a small decrease.

**Page 14 and elsewhere** Where estimates might be negative it is better to separate the limits with the word to or some other punctuation mark to avoid confusion.

**Page 20** I think the word slum has become seen as disrespectful these days. I do not think anything would be lost by stopping the sentence after unplanned settlements.

# Points of more substance

## Language

I have already remarked on this above. Just for the authors' information I nearly always raise this point, I have not singled them out for special criticism. In this case I think the issue is more serious than usual. Looking at the list of countries in Figure 2 it is striking that the main colonial power for nearly all of them was Britain. Ethiopia, although invaded, was never a colony of a European power and Namibia was a protectorate of South Africa. This raises in my mind the question whether the absence of countries from Francophone Africa is an artefact of just accepting English language sources. Since the authors (see S3) chose to limit the search in the way they have done this means that we do not know whether such studies exist but were not translated or whether they do not exist.

Even more striking is the complete absence of the Americas. There is a specialist bibliographical database for Latin America and the Caribbean: LILACS `https://lilacs.bvsalud.org/`.

I would suggest that broadening the search to include at least French, Portuguese (which would also include Lusophone Africa) and Spanish would tell

us whether the gaps in coverage are real or artefactual.

### Using a Bayesian approach in health research

Given the dominance of frequentist thinking in health I suspect the authors are going to have to justify the absence of $p$–values. In particular this is because they are still interpreting central estimates as reportable even when they fall within the credible interval. In general frequentist authors would be criticised for interpreting estimates which fall within the confidence interval. I am not really sure as to how best to justify this. Just for information I usually use frequentist methods although I find both long-fun frequency and subjective probability philosophically unappealing.

One advantage often expressed for a Bayesian approach in meta–analysis is that it avoids having to treat as known what are in fact estimates. On particular this applies to $\tau^2$ which in the frequentist paradigm is treated as known. I miss therefore any attempt to show values of $\tau^2$ which I assume must be available although I have never used the package (brms) which the authors have used.

### Repeated observation of countries

From Figure 2 it is clear that several countries provide more than one national survey or more than one sub–national survey. Observations within country must be presumed to be correlated and this reduces the between observation variability. Have I missed this point or has it been taken into account in the meta–analysis and meta–regressions?

## Summary

Some points for clarification and some thoughts about world coverage and about a technical issue with the meta-analysis.

Michael Dewey

## References

V C Bachelet, M S Navarrete, C Barrera-Riquelme, V A Carrasco, M Dallaserra, R A Díaz, A A Ibarra, F J Lizana, N Mezza-Ducaud, M G Saavedra, C Tapia-Davegnoa, A F Vergara, and J Villanueva. A multiyear systematic survey of the quality of reporting for randomised trials in den-

tistry, neurology, and geriatrics published in journals of Spain and Latin America. *BMC Medical Research Methodology*, 21(153), 2021.

M Egger, T Zellweger-Zähner, M Schneider, C Junker, C Lengeler, and G Antes. Language bias in randomised controlled trials published in English and German. *Lancet*, 350:326–329, 1997.

G Grégoire, F Derderian, and J Le Lorier. Selecting the language of the publications included in a meta-analysis: is there a tower of Babel bias? *Journal of Clinical Epidemiology*, 48(1):159–163, 1995.

J L Jackson, A Kuriyama, A Anton, A Choi, J-P Fournier, A-K Geier, F Jacquerioz, D Kogan, and R Sun. The accuracy of Google Translate for abstracting data from non–English–language trials for systematic reviews. *Annals of Internal Medicine*, 171:677–679, 2019.

P Jüni, F Holenstein, J Sterne, C Bartlett, and M Egger. Direction and impact of language bias in meta–analyses of controlled trials: empirical study. *International Jounal of Epidemiology*, 31:115–123, 2002.

K Konno, M Akasaka, C Koshida, N Katayama, N Osada, R Spake, and T Amano. Ignoring non–English–language studies may bias ecological meta–analyses. *Ecology and Evolution*, 10:6373–6384, 2020.

D Moher, P Fortin, A R Jadad, P Jüni, T Klassen, J Le Lorier, A Liberati, K Linde, and A Penna. Completeness of reporting of trials published in languages other than English: implications for conduct and reporting of systematic reviews. *Lancet*, 347:363–366, 1996.

B Nussbaumer-Streit, I Klerings, A I Dobrescu, E Persad, A Stevens, C Garrity, C Kamel, L Affengruber, V J King, and G Gartlehner. Excluding non–english publications from evidence–syntheses did not change conclusions: a meta–epidemiological study. *Journal of Clinical Epidemiology*, 118:42–54, 2020.
