# Supplementary material for: Urban and rural prevalence of tuberculosis in low- and middle-income countries: A systematic review and meta-analysis
Source: PLoS Med. 2026 Apr 6;23(4):e1004779. doi: 10.1371/journal.pmed.1004779 (PMC13068319; doi:10.1371/journal.pmed.1004779)
Supplement: Attachment — Submitted filename: Reviewer report_Plos medicine.docx [file pmed.1004779.s015.docx]

**Dear Authors,**

Thank you for the opportunity to review your manuscript, *“Urban and rural prevalence of tuberculosis in low- and middle-income countries: a systematic review and meta-analysis.”* This is a timely and important study that addresses critical gaps in our understanding of TB epidemiology across diverse settings. The manuscript is well-structured and provides valuable insights into urban–rural differences in TB prevalence. I have provided detailed comments and suggestions below aimed at clarifying methods, improving consistency, and enhancing the overall clarity and impact of the manuscript.

**Title:**
The review aims to assess TB prevalence in low- and middle-income countries (LMICs). However, a few included studies are from upper-middle-income countries such as China and Mongolia. According to the World Bank classification, these countries are upper-middle-income, not low- or lower-middle-income. I strongly recommend that the authors clarify how country income status was defined. Alternatively, consider including studies from upper-middle-income countries and modifying the title to reflect broader economic settings, e.g., *“Urban and rural prevalence of tuberculosis across diverse economic settings: a systematic review and meta-analysis.”*

**Abstract:**
In the Methods section, the authors mention including TB prevalence studies from LMICs between 1993 and 2024. However, in the Results subsection, the included surveys are reported as conducted between 2000 and 2019. Please clarify this discrepancy for readers.

**Background:**
The Background provides useful context but requires refinements to improve clarity and alignment with the study's aims:

1. Clearly define “LMICs” and indicate whether upper-middle-income countries (e.g., China) were included. If so, justify this early.
2. Explicitly highlight the knowledge gap, although national surveys report urban–rural differences, no global quantitative estimate exists.
3. Organize urban and rural determinants more clearly (urban factors together, rural factors together, then migration) to improve readability.
4. Provide a clearer rationale for quantifying urban–rural TB differences in terms of policy, resource allocation, and End TB targets.

**Methods:**

1. The manuscript mentions including studies published between 1 January 1993 and 1 January 2024. Please clarify why this period was selected and ensure consistency across sections.
2. Confirm that all included studies define adults as 15 years and above. If some studies use 18+, report these definitions.
3. Using WHO regions may be less informative than income-based stratification, which aligns more closely with your title and TB prevalence objectives.
4. I commend the authors for applying this method. It appropriately accounts for between-study variability, incorporates uncertainty, and allows inclusion of relevant covariates. This is a methodological strength.

**Results:**

1. Correct the total number of excluded studies in the PRISMA flow chart; currently, it shows 86, but it should be 155.
2. Correct minor typos and missing full stops (e.g., line 214).
3. The interpretation is not clear to readers. The Western Pacific region is reported with PR 0.64 (95% CrI: 0.45–0.89). Clarify that this indicates **urban TB prevalence is 36% lower than rural prevalence** for clearer interpretation.
4. Upper-middle-income countries are included despite the title claiming LMICs. Either remove these countries or include other upper-middle-income countries and modify the title accordingly. I recommend reanalysing subgroup analyses by **World Bank income classification**, which aligns with TB prevalence objectives. Use WHO regions only if focusing on geographic or regional patterns.

**Discussion (General Comments):**

The discussion is comprehensive, clearly linking urbanization, demographic changes, and TB prevalence. Regional heterogeneity and social determinants are well-addressed, and policy implications are highlighted. Minor improvements could enhance clarity: group similar points to reduce repetition and explicitly connect demographic trends to meta-analysis findings. Limitations are acknowledged, but a brief discussion of how they might affect urban–rural comparisons would strengthen interpretation. Overall, the discussion effectively conveys the public health relevance of the findings.
