# Supplementary material for: Urban and rural prevalence of tuberculosis in low- and middle-income countries: A systematic review and meta-analysis
Source: PLoS Med. 2026 Apr 6;23(4):e1004779. doi: 10.1371/journal.pmed.1004779 (PMC13068319; doi:10.1371/journal.pmed.1004779)
Supplement: Attachment — Submitted filename: 2025-12-02_urban-rural-tb_response.docx [file pmed.1004779.s017.docx]

**Prof Peter MacPherson**
MBChB(Hons) MPH MRes DTM&H DipHIV MFPH PhD

Professor of Global Public Health
Head of Public Health
[peter.macpherson@glasgow.ac.uk](mailto:peter.macpherson@glasgow.ac.uk)

18/12/2025

Dear Andreia Cunha,

**RE: Urban and rural prevalence of tuberculosis in low- and middle-income countries: a systematic review and meta-analysis" (PMEDICINE-D-25-03336R1)**

I write on behalf of my co-authors to thank the reviewers for their careful review, and appreciate the opportunity to respond with a revised submission. Please see point-by-point responses below.

In response to recommendations from reviewers, we have expanded our systematic literature search to include non-English reports on prevalence surveys (French, Spanish, Portuguese). We have also updated our search to 14^th^ October 2025. Furthermore, as WHO published updated global TB epidemiology estimates in November 2025, we have updated our modelled projections of overall, and country-specific urban and rural TB burden to 2024. With these additions, we have made other minor changes to reporting of methods and results throughout; these are indicated with track changes.

With these changes, we believe that the manuscript is considerably strengthened.

Yours Sincerely,

Peter MacPherson (on behalf of the authors)

**Reviewer 1**

**Comment #1.1:** The authors acknowledge the limitation that urban-rural distinction may mean different things in different regions and countries. An additional limitation in this regard may be that population denominators for tuberculosis prevalence estimates are generally based on census data and where those are not recent, on intercensal growth rates. These intercensal growth rates may not reflect recent changes, for example because due to rapid urbanization and urban-rural migration, the growth of urban populations is steeper than based in intercensal interpolation. I think this potential limitation should be acknowledged as well.

**Author’s Response:**

Thank you for this comment. There are two sets of population denominators relevant to this point. Firstly, for the prevalence surveys themselves, usually (and particularly in national prevalence surveys) census surveys of the study population were undertaken by prevalence survey teams to generate sampling frames for the prevalence study. Secondly, where we construct epidemiological methods to estimate time-trends in country-specific urban and rural prevalence we use UN Population Prospects and World Bank estimates. Whilst these are generally recognised to be the most accurate population estimates available, we agree that these may misestimate urban population growth, in particular in areas of recent, rapid urbanisation.

Line 954-956: *“To estimate urban and rural TB burden, we used population estimates from the United Nations and World Bank; these may misestimate population growth, and particular rates of urbanisation.”*

**Comment #1.2:** The prevalence estimates used in the meta-analyses were as much as possible based on adjustments made based on WHO-recommended analyses. A key adjustment here is inverse probability weighting for age-sex specific non-participation rates. As participation rates in TB prevalence surveys tend to be particularly low among young men who are often also the age-sex stratum with the highest TB prevalence, these adjustments have had large (upward) impact on the prevalence estimates from national TBPS for some countries, e.g. Nigeria and Philippines. However, the implicit underlying assumption that non-participation is random may not hold true, e.g. due to healthy worker bias (those who are healthy are at work and don't show up for the survey). As young men will be often be the ones that migrate to urban areas this may bias the proportion TB that is urban versus rural. I would therefore suggest that the investigators add a sensitivity analysis where the unadjusted estimates from
the surveys are used rather than the adjusted estimates.

**Author’s Response:**

We agree with the reviewer that prevalence estimates are sensitive to differential rates of participation by particular groups, with the greatest rates of non-participation seen among younger men. Indeed, we explicitly accounted for this by basing our primary analysis on meta-analysis of prevalence estimates adjusted for non-participation and sampling bias using the WHO-recommended “model 3 approach”, where available. We describe in Lines 165-167 and illustrate our hierarchical-approach to inclusion of available survey-specific estimates in meta-analysis S4 Fig.

We however disagree with the reviewer that meta-analysis of unadjusted prevalence estimates would mitigate this. To explain: unadjusted prevalence estimates are systematically biased away from “true” population prevalence due to lower participation of some groups; meta-analysis of these unadjusted estimates would not address sampling bias, and would likely produce more biased summary estimates. Furthermore, national prevalence surveys (which due to their size contribute the greatest weight in meta-analysis) frequently do not provide crude prevalence estimates in reports, as they recognise that these are systematically biased. Therefore, if we were to undertake meta-analysis of unadjusted prevalence estimates, we would likely exclude a substantial fraction of the highest quality data from national surveys.

Therefore, for the reasons above, we are reluctant to undertake meta-analysis of unadjusted prevalence estimates as we feel this is less methodologically rigorous, and would be likely to introduce substantially more biased estimates into the manuscript, which we would find difficult to justify. Should the Editor feel strongly that this is required, we would be happy to add a small section to the supplemental material to report this.

**Comment #1.3:** If I understand it correctly, the Statistical modelling of incidence and case detection was based on WHO estimates of country-specific TB incidence and case detection rates where the urban-rural distribution of TB prevalence was derived from the meta-analysis. The WHO estimates of TB incidence and case detection rate are however not independent of the prevalence survey results, for many countries they are directly based on them. Should that interdependence be considered a problem

**Author’s Response:**

Thank you for this important comment. We acknowledge that WHO estimates of TB incidence and case detection are, in some countries (but by no means all), informed in part by prevalence survey data. In our analysis, however, these data sources play distinct roles. WHO incidence and case detection estimates are used to inform our modelled national TB prevalence trajectories using a Bayesian multivariate model with explicit assumptions about disease duration. We also use Bayesian partial pooling to share information between countries, allowing estimates for data-sparse settings to be informed by broader regional and temporal patterns, thereby reducing reliance on any single country’s prevalence surveys and mitigating potential circularity with WHO inputs.

Separately, the prevalence survey data are used to estimate relative urban–rural differences in TB prevalence, expressed as prevalence ratios, which are not directly estimated by WHO. These urban–rural contrasts are then applied within the epidemiological model to infer subnational burden over time. Thus, although prevalence surveys may contribute to WHO estimates, they are not reused to estimate the same quantities: WHO data inform national temporal trends, while collected prevalence survey data inform geographical contrasts within countries. Uncertainty from both components is fully propagated through the Bayesian framework. Nevertheless, we have added text to the discussion to highlight this potential limitation:

Lines 423-428: *“Approaches used by WHO to estimate TB incidence and case detection make use of prevalence survey data in some settings, which could create dependences between data sources if we also used these survey data for our analysis. For this reason, we only used prevalence survey data to estimate urban–rural contrasts, and relied on WHO data to estimate overall prevalence levels; together with Bayesian partial pooling across countries, we believe this is unlikely to materially bias subnational TB burden estimates.”*

**Comment #1.4:** An associated potential limitation is that for a number of countries in the analyses, the WHO estimates of TB incidence are unchanged over the 19-year period (Fig S10). That seems odd, and may be a reflection of lack of data or other limitations on the part of WHO. I believe that the limitations of the WHO estimates should be mentioned as well.

**Author’s Response:**

That you for this comment, and the reviewer is correct that in a small number of countries (specifically Nigeria, Bangladesh, and North Korea), WHO incidence estimates are unchanged, with associated uncertainty being wide. This is due to a lack of data reported to WHO by these countries to permit more precise estimates.

For this revision, we updated our search to 14^th^ October 2025, including one additional national TB prevalence survey in Cambodia done in 2024. We therefore also updated our analysis of TB burden, using updated global and national TB incidence and case detection estimates published in the Global TB Report by WHO in November 2025. In these most recent estimates, WHO has removed all incidence and case detection estimates for 2000-2024 for North Korea, stating “*estimates of TB incidence are currently under review*”. We have therefore removed North Korea data from our models estimating urban and TB burden. We retain Bangladesh and Nigeria in estimates, as WHO continues to report incidence and case detection estimates for these countries.

We have additionally added text to the Discussion to highlight this limitation:

Lines 419-422: “*To estimate numbers of people with prevalent TB and temporal trends, we relied on WHO incidence and case detection data combined with assumptions about disease duration recognising that for Nigeria and Bangladesh, WHO estimates of incidence are uncertain.”*

**Comment #1.5:** Line 230: "%" seems missing

**Author’s Response:**

Thanks. We have corrected this.

**Comment #1.6:** Table 1, bottom row. Is this about symptom screening ONLY used for sputum eligibility (i.e. no chest X-ray screening)? In my view it should, because surveys that only did symptom screening will not detect asymptomatic TB and studies have suggested that asymptomatic TB is more common in urban settings.

**Author’s Response:**

Thanks. Yes, this stratification compared studies where screening was done on the bases of symptoms alone vs. chest X-ray +/- symptom screening. We have clarified in the table. We are unaware of data showing that asymptomatic TB is more common in urban areas.

**Comment #1.7:** Discussion, lines 327-330: "(…) as emphasised by the absence of association with urbanisation indicators in meta-regression". I may have missed it but where exactly is this in the Results?  Table 1 only shows one such indicator (Percent of population in urban areas), I did not see other important indicators such as population density.

**Author’s Response:**

Apologies. By “urbanisation” we meant the proportion of the population that lives in urban areas, and how this changed over the study period, as analysed in our meta-regression model. We did not have spatially-resolved data to permit investigation of the association between population density and TB prevalence, as this would require individual-level geolocated data from all prevalence surveys, which is challenging to obtain. Table 1 describes how this variable is defined: *“Percent of population in urban areas (per % point increase)”*. We have revised the text to state:

Lines 363-366: *“These findings suggest that demographic and epidemiological processes are acting in concert, with urban growth amplifying—but not solely determining, as emphasised by the absence of association with urban-population fraction in the meta-regression—the increasing concentration of TB in cities.”*

**Reviewer 2**

**Comment #2.1:** The review aims to assess TB prevalence in low- and middle-income countries (LMICs). However, a few included studies are from upper-middle-income countries such as China and Mongolia. According to the World Bank classification, these countries are upper-middle-income, not low- or lower-middle-income. I strongly recommend that the authors clarify how country income status was defined. Alternatively, consider including studies from upper-middle-income countries and modifying the title to reflect broader economic settings, e.g., "Urban and rural prevalence of tuberculosis across diverse economic settings: a systematic review and meta-analysis."

**Author’s Response:**

Thank you. We were somewhat confused by this comment, as the reviewer seems to be suggesting that “upper-middle income countries” are not middle-income countries.

The [World Bank Country and Lending Groups](https://datahelpdesk.worldbank.org/knowledgebase/articles/906519-world-bank-country-and-lending-groups) has four levels: “lower income”; “lower-middle income”; “upper-middle income”; and “high income”. Note that the World Bank recognises that “countries” and “economies” can be used interchangeably. As is standard, we defined “low- and middle-income countries” to be all these groups excluding “high income”, and thus as per the title, our study includes “low- and middle-income countries”. We have added a sentence to the Methods to clarify this inclusion criteria.

Lines 98-103: “*We included studies published between 1st January 1993 (the year when WHO declared TB to be a public health emergency of international concern) and 14^th^ October 2025 that reported the prevalence of pulmonary TB in both rural and urban adult populations (15 years or older) in LMICs (as defined by the World Bank country groups “lower income”, “lower-middle income”, and “upper-middle income” [14]).”*

**Comment #2.2:** Abstract: In the Methods section, the authors mention including TB prevalence studies from LMICs between 1993 and 2024. However, in the Results subsection, the included surveys are reported as conducted between 2000 and 2019. Please clarify this discrepancy for readers.

**Author’s Response:**

We systematically searched the literature from 1^st^ January 1993 and 14^th^ October 2025 (in our updated search for this revision). Prevalence studies contributing data to the review were conducted between 2000 and 2024 (i.e., no studies meeting inclusion criteria were conducted between 1993 and 1999). This is not a discrepancy, rather a description of the study methods and results.

**Comment #2.3:** Background: Clearly define "LMICs" and indicate whether upper-middle-income countries (e.g., China) were included. If so, justify this early.

**Author’s Response:**

Please see response to point 2.1 above. We included World Bank country groupings of “lower income”; “lower-middle income”, and “upper-middle income”. We did not include “high income” countries. Therefore, we did include all middle income countries. Text edits made to clarify these inclusion criteria are given under comment #2.1.

**Comment #2.4:** Background: Explicitly highlight the knowledge gap, although national surveys report urban-rural differences, no global quantitative estimate exists.

**Author’s Response:**

Lines: 111-112:

Thank you. We state this in Lines 85-87:

*“Although some national TB prevalence surveys have found differences in TB prevalence between urban and rural areas (e.g. [11]), the magnitude of this difference has not been systematically quantified, and there is little understanding of country and regional patterns and determinants.”*

**Comment #2.5:** Background: Organize urban and rural determinants more clearly (urban factors together, rural factors together, then migration) to improve readability.

**Author’s Response:**

Thank you. In the Background (Lines 77-84), we grouped discussion of urban and rural determinants of TB together:

*“Understanding variation in TB burden both between and within countries and regions is essential for developing effective public health strategies to address TB determinants and accelerate TB care and prevention [3]. Urban and rural environments differ substantially in the factors that shape TB transmission, healthcare access, and health-seeking behaviours [4]. Urban areas may experience higher TB prevalence due to greater population density, higher rates of HIV infection, and increased exposure to crowded congregate settings and poor outdoor air quality [5]. In contrast, people living in rural areas can face barriers such as limited healthcare access, delayed diagnosis, and treatment challenges, as well as higher rates of indoor air pollution [6,7].”*

**Comment #2.6:** Background: Provide a clearer rationale for quantifying urban-rural TB differences in terms of policy, resource allocation, and End TB targets

**Author’s Response:**

Thank you. We have edited Lines 88-91 to emphasise this point:

*“Therefore, we conducted a systematic review and meta-analysis using data from national and sub-national TB prevalence surveys conducted in LMICs. We compared urban and rural TB prevalence and estimated burden and secular trends of urban and rural TB within LMICs and WHO regions to inform targeted resource allocation and public health approaches to TB elimination.”*

**Comment #2.7:** Methods: The manuscript mentions including studies published between 1 January 1993 and 1 January 2024. Please clarify why this period was selected and ensure consistency across sections.

**Author’s Response:**

Thank you. We started our inclusion period in 1993 as this is when WHO declared tuberculosis to be a public health emergency of international concern, and substantial global and national increases in resourcing began to be committed to addressing this pandemic. We have added text to explain this in the Methods:

Lines 98-105: “*We included studies published between 1^st^ January 1993 (when WHO declared TB to be a public health emergency of international concern) and 14^th^ October 2025 that reported the prevalence of pulmonary TB in both rural and urban adult populations (15 years or older) in LMICs (as defined by the World Bank country groups “lower income”, “lower-middle income”, and “upper-middle income” [14]).”*

As per response to comment #2.2 above, there is no discrepancy between inclusion criteria dates and dates when included studies were conducted. This is simply a description of the study methods and results.

**Comment #2.8:** Methods: Confirm that all included studies define adults as 15 years and above. If some studies use 18+, report these definitions.

**Author’s Response:**

Many thanks for this comment. We have gone back and checked age inclusion criteria for all surveys. All but three of the 47 surveys included adults aged 15 years or older. One survey included people aged 10 years or older, one 12 years, and aged 14 years or older. In all three studies, the numbers in these age groups <15 years were very small. These studies remain eligible in accordance with our protocol, where numbers of adults 15 years or above predominate, and it is not possible to exclude participants in the younger age strata from prevalence estimates. We have added text to the methods to clarify this:

Lines 98-105: “*We included studies published between 1^st^ January 1993 (the year when WHO declared TB to be a public health emergency of international concern) and 14^th^ October 2025 that reported the prevalence of pulmonary TB in both rural and urban adult populations (15 years or older, or where the majority of participants where 15 years or older and it was not possible to disaggregate prevalence estimates for people younger than 15 years) in LMICs (as defined by the World Bank country groups “lower income”, “lower-middle income”, and “upper-middle income” [14]).”*

**Comment #2.9:** Methods: Using WHO regions may be less informative than income-based stratification, which aligns more closely with your title and TB prevalence objectives.

**Author’s Response:**

Thank you for this thoughtful suggestion. We agree that national income classification can, in some contexts, provide useful contextual information; however, for this analysis we believe WHO regional groupings offer a more epidemiologically meaningful stratification for interpreting variation in TB prevalence.

Firstly, WHO regions reflect long-established differences in TB epidemiology, health-system organisation, diagnostic capacity, and broader demographic and socio-political contexts that are relevant to population-based TB prevalence survey implementation and outcomes. These factors often align more closely with how TB programmes are structured, supported, and evaluated internationally. By contrast, World Bank income categories group together countries with markedly heterogeneous TB burdens, epidemic trajectories, and health-system capacities, and they change over time based on economic indicators that are not themselves necessarily determinants of TB epidemiology.

Second, our primary objective was to compare urban–rural TB prevalence differences across settings with shared epidemiological profiles, namely countries and WHO regions. WHO regions provide stable, policy-relevant strata that are routinely used for global TB guidance, reporting and programme benchmarking.

For these reasons we have retained WHO regional stratification in the main analysis. To acknowledge the reviewer’s point, we now report country income classifications in S6 Table.

**Comment #2.10:** Methods: I commend the authors for applying this method. It appropriately accounts for between-study variability, incorporates uncertainty, and allows inclusion of relevant covariates. This is a methodological strength.

**Author’s Response:**

Thank you.

**Comment #2.11:** Results: Correct the total number of excluded studies in the PRISMA flow chart; currently, it shows 86, but it should be 155.

**Author’s Response:**

Thank you for identifying this. We have updated the PRIMSA flowchart to reflect the extended search period (to 14^th^ October 2025) and the inclusion of additional databases containing non-English journals.

**Comment #2.12:** Results: The interpretation is not clear to readers. The Western Pacific region is reported with PR 0.64 (95% CrI: 0.45-0.89). Clarify that this indicates urban TB prevalence is 36% lower than rural prevalence for clearer interpretation.

**Author’s Response:**

Thank you for the suggestion to clarify the interpretation of regional prevalence ratios. We have added text to emphasise relative differences between urban and rural prevalence in percentage terms.

Lines 244-251: *“In our main model, which excluded two studies from the Eastern Mediterranean Region with disparate crude estimates, the pooled urban-to-rural prevalence ratio for bacteriologically-confirmed TB was 1.07, (95% CrI: 0.87, 1.30) (Figure 2). This is equivalent to prevalence rates being 7% higher in urban settings, but with an uncertainty interval including 0%. The magnitude and direction of the urban–rural difference varied by WHO region. The African Region showed higher prevalence in urban settings* *(PR 1.24, 95% CrI: 0.95, 1.56), equivalent to 24% higher prevalence in urban areas), whereas in the Western Pacific Region prevalence was higher in rural settings (PR 0.70, 95% CrI: 0.49, 0.94, equivalent to 30% higher in rural areas). Estimates for the South-East Asia Region indicated little evidence of a systematic difference (**PR 0.84, 95% CrI: 0.65, 1.07).”*

**Comment #2.13:** Results: Upper-middle-income countries are included despite the title claiming LMICs. Either remove these countries or include other upper-middle-income countries and modify the title accordingly. I recommend reanalysing subgroup analyses by World Bank income classification, which aligns with TB prevalence objectives. Use WHO regions only if focusing on geographic or regional patterns.

**Author’s Response:**

Thank you for these comments, which have been addressed in response to points #2.1, #2.3 and #2.9 above. Specifically, we included “low- and middle-income countries”, as per our title and methods.

**Comment #2.14:** Discussion: The discussion is comprehensive, clearly linking urbanization, demographic changes, and TB prevalence. Regional heterogeneity and social determinants are well-addressed, and policy implications are highlighted. Minor improvements could enhance clarity: group similar points to reduce repetition and explicitly connect demographic trends to meta-analysis findings. Limitations are acknowledged, but a brief discussion of how they might affect urban-rural comparisons would strengthen interpretation. Overall, the discussion effectively conveys the public health relevance of the findings.

**Author’s Response:**

Thank you. We have revised the Discussion, including the limitations paragraphs, to incorporate these recommendations.

**Reviewer 3**

**Comment #3.1:** Page 5 The authors have chosen to only include studies published in English. The message I draw from Gregoire et al. (1995); Moher et al. (1996); Egger et al. (1997); Juni et al. (2002); Konno et al. (2020); Nussbaumer-Streit et al. (2020); Bachelet et al. (2021) is that bias is possible although it is hard to predict in advance the extent or direction of it. I would not necessarily commit the authors to translate everything but at least we should know what we are missing.

**Author’s Response:**

Thank you. We agree that this is important. In this revised version, as well as extending our search to 14^th^ October 2025 (which identified one additional WHO-supported national TB prevalence survey from Cambodia done during 2024), we systematically searched three databases with coverage of journals published in Spanish, French, and Portuguese (Africa Index Medicus, LILACS, and SciELO). This identified 2,552 manuscripts, which were translated using DeepL (see response to comment #3.2 below), and screened by 2 authors. Four non-English manuscripts underwent full-text review following translation by DeepL, and none met eligibility criteria for inclusion. As WHO maintains a repository of national prevalence surveys, we are confident that no WHO-supported foreign-language national surveys have been excluded.

We have updated the study methods, results, and figures to describe this. We have additionally modified the discussion to state that, despite searching databases covering these three languages focused on high TB burden regions, there is a paucity of evidence, particularly from the WHO Region of the Americas.

**Comment #3.2:** Page 5 The authors refer to limited translation resources. In a small study Jackson et al. (2019) reported good results in evidence synthesis using Google Translate. DeepL is another possibility.

**Author’s Response:**

That you. We experimented with ChatGPT 5.1, Apple Translate, and DeepL, with DeepL ultimately performing best in terms of ease of use and accuracy. We used DeepL for translation of non-English language studies, as described above.

**Comment #3.3:** Page 6 That is a very clear report of the screening and abstraction process and the precautions taken against bias.

**Author’s Response:**

Thank you.

**Comment #3.4:** Page 7 Does the statement ‘potential predictors included’ mean that there were others which have not been reported? This is important if others were considered but finally discarded.

**Author’s Response:**

Thank you. We apologise for our imprecise language here. We only examined these predictors. We have modified this sentence to correct.

Lines 149-150: “*Predictors included the proportion of the study country population who resided in urban areas [17] and country-level TB incidence in the prevalence survey year [1].”*

**Comment #3.5:** Page 8 Do the authors mean a multivariate model for incidence and case detection data, that is to say, a model with a vector–valued outcome, or do they mean a multivariable model? They use multivariable correctly elsewhere so I assume multivariate is meant here.

**Author’s Response:**

Thank you for drawing attention to this terminology. In this section we do indeed mean a *multivariate* model, as incidence and case detection ratio were modelled jointly as correlated outcomes within a single Bayesian framework. We have revised the text to make this explicit and to avoid any potential confusion with multivariable regression.

Lines 190-192: *“To estimate the number and percentage of people with prevalent TB in urban and rural areas in study countries, and how these have changed between 2000 and 2024, we fitted a Bayesian multivariate model in which WHO TB incidence and case detection ratio were modelled jointly as correlated outcomes.”*

**Comment #3.6:** Page 9 Providing proper citation for R packages as here gives authors appropriate credit for their work. (Disclaimer: I am a CRAN author although of different packages.)

**Author’s Response:**

Thanks. We agree, and cite Stan and R packages:

Lines: 198-202: *“Models were fit using the brms interface (version 2.22.0) [22] to Stan (version 2.37.0) [23]. We assessed model convergence using* $\hat{R}$ *statistics, effective sample size measures, trace plots of chains, and posterior predictive plots. Model predictions and summaries used the tidyverse [24] and tidybayes [25]. All analyses were conducted using R version 4.5.0 [26].”*

**Comment #3.7:** Page 10, Figure 1 Studies assessed 201, studies excluded 86, studies included 45, 201−86 ̸= 45. Is this explained by the slightly cryptic note in the caption about multiple reporting? If so how was the decision made as to which to include?

**Author’s Response:**

We apologies for this inadvertent mistake. We have revised the search to include non-English studies (Portuguese, Spanish, and French) and have extended the search until 14^th^ October 2025. We have carefully revised Figure 1 to ensure all numbers are correct. Additionally, we note the reviewer’s comment about multiple publications reporting on single surveys. This did happen on a few occasions (e.g., Ministry of Health National Prevalence Survey report and journal manuscript). We handled this by grouping and reviewing all related publications together, and extracting data from the combined set. We have added text to the methods to describe this:

Lines 127-129: *“Where there were multiple publications relating to one prevalence survey, we grouped these, reviewed all together, and extracted data from the combined set.”*

**Comment #3.8:** Page 12 There is a dilemma here between (a) choosing to use an established classification (WHO regions) which has advantages of being defined independently of the authors and (b) using a sensible but ad hoc one. I have no particular problem with most of the WHO regions but their idea of the Eastern Mediterranean is not mine. I do not see many common features between Sudan and Pakistan. I think most people would say Sudan is in Africa and Pakistan is in South Asia. The inclusive approach would be to allocate them to Africa and South East Asia respectively and explain why the WHO regions have had to be modified.

**Author’s Response:**

Thank you, and we agree that this is an important comment, and one that we wrestled with whilst conducting the review. We have also addressed some aspects of this comment in points #2.1, #2.3, #2.9, and #2.13 above.

We appreciate the reviewer’s careful engagement with the choice of regional classification and agree that any regional grouping necessarily involves trade-offs between conceptual coherence and analytic consistency. After careful consideration, we have retained the WHO regional classification throughout the analysis.

Our primary reason for doing so is that WHO regions represent an externally defined, policy-relevant framework that underpins global TB surveillance, reporting, and programme planning. Although individual countries within a region may differ epidemiologically, the use of an established classification avoids ad-hoc reallocation of countries based on authors’ judgement, which risks introducing subjectivity and reducing reproducibility. In particular, reassigning countries such as Sudan or Pakistan to alternative regions would constitute a post hoc modification of a standard global framework and could undermine comparability with WHO TB estimates and prior literature.

We recognise the reviewer’s concern regarding the small number of studies from the Eastern Mediterranean Region and their epidemiological heterogeneity. In response, we have revised the analytical approach in two ways.

First, in meta-analysis we present pooled regional estimates only for regions with sufficient data (Africa, South-East Asia, and Western Pacific). For the two studies from the Eastern Mediterranean Region, we report study-level estimates without generating a pooled regional summary, thereby avoiding over-interpretation of a heterogeneous and sparsely populated category. As an exploratory analysis, we reassigned the two countries from the Eastern Mediterranean Region to alternative WHO regions based on geographic convention (Sudan to the African Region; Pakistan to the South-East Asia Region) and refitted the main-effects model. Posterior summaries of the heterogeneity parameters were essentially unchanged (see point #3.14 below). The posterior median study-within-country standard deviation remained τ ≈ 0.10 (τ² ≈ 0.011), and the between-country variance was similarly stable (posterior median ≈ 0.15). The total random-effects variance was nearly identical to that observed in the primary analysis. The close agreement of τ and τ² across the primary and recoded models indicates that estimates of between-study and between-country heterogeneity are robust to alternative regional assignments for these two countries. This further supports our decision to retain WHO regional classifications in the main analysis and to address sparsity and heterogeneity in the Eastern Mediterranean Region through model structure and reporting, rather than through ad hoc reallocation of countries.

Second, in the updated epidemiological modelling of TB burden over time, we include a random effects structure with surveys nested within countries to estimate country-specific time trends. This allows Sudan and Pakistan to contribute to the modelling and burden estimation, whilst ensuring that inference is driven by country-level data, rather than by assumptions of regional homogeneity.

We have clarified this rationale in the Methods and Discussion, emphasising that WHO regional groupings were retained for transparency and comparability, while analytic strategies were adapted to account for sparse data and heterogeneity in specific regions.

Lines 190-196: *“**To estimate the number and percentage of people with prevalent TB in urban and rural areas in study countries, and how these have changed between 2000 and 2024, we fitted a Bayesian multivariate model in which WHO TB incidence and case detection ratio were modelled jointly as correlated outcomes. The model included random effects for surveys nested within country, allowing country-specific and time-varying estimates, including for countries with sparse regional representation. We combined these estimates with assumptions about the duration of treated and untreated TB (stratified by smear status) [21] and the distribution of urban and rural populations (S5 Text) [17].”*

**Comment #3.9:** Page 12 Comparison between the regions would be clearer if we also saw estimates of the relative prevalence ratio with a credible interval.

**Author’s Response:**

We thank the reviewer for this suggestion. We now report explicit between-region contrasts derived from the posterior distributions of the regional effects, expressed as ratios of urban–rural prevalence ratios with 95% credible intervals. We have added a brief summary to the Results section to report these comparisons.

Lines 252-255: “*Comparing between WHO regions, the urban–rural prevalence ratio was higher in the African Region than in the Western Pacific Region (ratio of prevalence ratios 1.90, 95% CrI: 1.20, 2.83) and higher than in the South-East Asia Region (1.54, 95% CrI: 1.07, 2.14), while differences between South-East Asia and the Western Pacific were less certain (1.25, 95% CrI: 0.84, 1.81).”*

**Comment #3.10:** Page 14 Although it is perfectly true that 2.2% credible interval -2.5 to 8.0 is consistent with a small increase it is also consistent with a small decrease.

**Author’s Response:**

Thank you. In our revised analysis, which include data from one additional national prevalence survey (Cambodia 2024), our revised estimate for the annual change in the urban-to-rural TB prevalence ratio is 2.2% (95% CrI: -1.7%, 6.7%) per year. We have added text to emphasise that this estimate has substantial uncertainty.

**Comment #3.11:** Page 14 and elsewhere. Where estimates might be negative it is better to separate the limits with the word to or some other punctuation mark to avoid confusion.

**Author’s Response:**

Thanks. We believe that PLOS Medicine uses commas to separate limits, and have updated the text throughout to this style.

**Comment #3.12:** Page 20 I think the word slum has become seen as disrespectful these days. I do not think anything would be lost by stopping the sentence after unplanned settlements.

**Author’s Response:**

Thank you for raising this. We note that WHO continues to use the word “slum” as recently as 31^st^ October 2025 in its most recent global strategy for urban health (<https://www.who.int/news/item/31-10-2025-who-calls-for-a-new-era-of-strategic-urban-health-action-with-global-guide-to-unlock-healthy-prosperous-and-resilient-societies>). Nevertheless, we appreciate the sensitivities here and have removed this text.

**Comment #3.13:** Language: I have already remarked on this above. Just for the authors’ information I nearly always raise this point, I have not singled them out for special criticism. In this case I think the issue is more serious than usual. Looking at the list of countries in Figure 2 it is striking that the main colonial power for nearly all of them was Britain. Ethiopia, although invaded, was never a colony of a European power and Namibia was a protectorate of South Africa. This raises in my mind the question whether the absence of countries from Francophone Africa is an artefact of just accepting English language sources. Since the authors (see S3) chose to limit the search in the way they have done this means that we do not know whether such studies exist but were not translated or whether they do not exist. Even more striking is the complete absence of the Americas. There is a specialist bibliographical database for Latin America and the Caribbean: LILACS <https://lilacs.bvsalud.org/>. I would suggest that broadening the search to include at least French, Portuguese (which would also include Lusophone Africa) and Spanish would tell us whether the gaps in coverage are real or artefactual.

**Author’s Response:**

Thank you for this comment, and we agree that this is an important issue. Please see also response to comment #3.1 above.

In this revised version, as well as extending our search to 14^th^ October 2025 (which identified one additional WHO-supported national TB prevalence survey from Cambodia), we systematically searched three databases with coverage of journals published in Spanish, French, and Portuguese (Africa Index Medicus, LILACS, and SciELO). This identified 2,552 manuscripts, which were translated (where required) using DeepL (see response to comment #3.2), and screened by two authors. Four manuscripts underwent full-text review following translation by DeepL, and none met eligibility criteria. As WHO maintains a repository of national prevalence surveys, we are confident that no WHO-supported foreign-language national surveys have been excluded.

We have updated the study methods, results, and figures to describe this. We have additionally modified the discussion the state that, despite searching databases covering these three languages focused on high TB burden regions, there is a paucity of evidence, particularly from the WHO Region of the Americas.

**Comment #3.14:** Using a Bayesian approach in health research. Given the dominance of frequentist thinking in health I suspect the authors are going to have to justify the absence of p–values. In particular this is because they are still interpreting central estimates as reportable even when they fall within the credible interval. In general frequentist authors would be criticised for interpreting estimates which fall within the confidence interval. I am not really sure as to how best to justify this. Just for information I usually use frequentist methods although I find both long-fun frequency and subjective probability philosophically unappealing. One advantage often expressed for a Bayesian approach in meta–analysis is that it avoids having to treat as known what are in fact estimates. On particular this applies to τ2 which in the frequentist paradigm is treated as known. I miss therefore any attempt to show values of τ2 which I assume must be available although I have never used the package (brms) which the authors have used.

**Author’s Response:**

Thank you for these thoughtful comments, which we appreciate. Our decision to use a Bayesian framework was motivated by practical inferential considerations.

Firstly, our primary aim was not hypothesis testing but estimation and uncertainty quantification. Bayesian models allow direct probabilistic statements about parameters of interest, including the probability that the urban-to-rural prevalence ratio exceeds unity, which aligns closely with our epidemiological objectives. In this framework, posterior means are valid summaries of the posterior distribution regardless of whether the 95% credible interval includes 1, and throughout, our interpretation is based on the full posterior rather than dichotomisation using arbitrary thresholds. We have edited text in the Discussion to emphasise this:

Lines 368-369: *“…prevalence was higher in urban areas, with an 90% posterior probability the urban-to-rural prevalence ratio exceeded one in 2024.”*

Secondly, a key motivation for our Bayesian approach was the coherent propagation of uncertainty across analytic stages. Posterior distributions from the meta-regression were carried forward into our epidemiological models estimating urban and rural TB burden over time. This would be difficult to achieve transparently within a frequentist framework, particularly when combining uncertainty from multiple sources.

Thirdly, as the reviewer notes, Bayesian meta-analysis avoids treating between-study heterogeneity as fixed once estimated. In our models, heterogeneity is explicitly represented through random effects with associated variance parameters that are themselves estimated and assigned priors, such that uncertainty in heterogeneity is fully propagated into posterior inference and downstream modelling. In our main effects model, the nested random-effects structure *(1 | study.country / survey_id)* (see point #3.15 below) yields two distinct variance components: a between-country variance and a between-survey-within-country variance. The closest analogue to the conventional meta-analytic heterogeneity parameter τ² is the posterior variance of the study-within-country random effects, i.e. $\tau^{2}\approx\mathrm{Var}(u_{\text{survey(country)}}),$ corresponding to sd_study.country:survey_id__Intercept² in brms. In this model, heterogeneity was modest at the study level and larger at the country level, indicating that residual variation was driven primarily by differences between countries rather than by variation across surveys conducted within the same country.

We further examined heterogeneity in our model that explicitly parameterised WHO region as a higher-level grouping, using a nested random-effects structure *(1 | WHO.region / survey_id)*. In this model, heterogeneity was partitioned into between-region and within-region components. Posterior summaries indicated that the between-region variance exceeded the within-region (study-level) variance, with wider uncertainty reflecting the small number of regions. This reallocation of variance is consistent with WHO regions capturing genuine structural differences in TB epidemiology and health-system context, rather than representing arbitrary aggregation.

Importantly, estimates of τ² remained stable across model specifications, supporting the robustness of our conclusions and the appropriateness of WHO regions as a policy-relevant stratification. These results are summarised in the figure below. To provide reassurance that priors on random effects are not overly influential, we also plot simulated prior distributions for the random intercept standard deviations (in purple).

Finally, to avoid confusion for readers more familiar with frequentist reporting, we have ensured that interpretation in the Results consistently refers to the magnitude and direction of effects, and to posterior probabilities, rather than implicit significance testing. We do not use p-values because they are not directly meaningful in the Bayesian framework and would be redundant given the posterior summaries presented.

**Comment #3.15:** Repeated observation of countries. From Figure 2 it is clear that several countries provide more than one national survey or more than one sub–national survey. Observations within country must be presumed to be correlated and this reduces the between observation variability. Have I missed this point or has it been taken into account in the meta–analysis and meta–regressions?

**Author’s Response:**

Thank you for this thoughtful comment, which we appreciated.

We agree that repeated observations within countries should be expected to be correlated and that failure to account for this could underestimate between-observation variability.

This issue has now been explicitly addressed in the revised manuscript. We updated our meta-analysis and meta-regression models to include nested random-effects structures, with prevalence surveys nested within countries *(1 | study.country / survey_id)*, and, in region-specific analyses, surveys nested within WHO regions *(1 | WHO.region / survey_id)*. These structures explicitly account for correlation among repeated observations from the same country or region.

Posterior summaries of urban–rural prevalence ratios from these updated models were essentially identical compared to our previous models without survey-level nesting.

Consistent with the findings reported in response to Comment #3.14, we found that heterogeneity was modest at the survey level and substantially larger at the country and regional levels. This indicates that residual heterogeneity is driven primarily by differences between countries or regions, rather than by variation across multiple surveys conducted within the same country. These results confirm that within-country correlation has been appropriately accounted for and does not materially affect our substantive conclusions.
